# Case report of the first cured patient with *Mycobacterium Chimaera* infection following cardiac valve replacement in the mainland of China

**DOI:** 10.1186/s13756-021-01003-9

**Published:** 2021-10-07

**Authors:** Di Lu, Chun-Hong Zhang, Li-Jiang Chen, Pei-Feng Jin, Xia-Fei Feng, Xi Zhou, Wei-Cong Huang, Jue Wang, Ying Dai, Yun Fu

**Affiliations:** 1 Department of Cardiac Surgery, First Affiliated Hospital, Wenzhou Medical University, Wenzhou, 325000 Zhejiang People’s Republic of China; 2 Department of Pharmacy, First Affiliated Hospital, Wenzhou Medical University, Wenzhou, Zhejiang People’s Republic of China; 3 Department of Clinical Laboratory, First Affiliated Hospital, Wenzhou Medical University, Wenzhou, Zhejiang People’s Republic of China; 4 Cardiac Intensive Care Unit, First Affiliated Hospital, Wenzhou Medical University, Wenzhou, Zhejiang People’s Republic of China

**Keywords:** *Mycobacterium chimaera* infection, Cardiac surgery, Molecular diagnosis, Metagenomic next-generation sequencing

## Abstract

**Background:**

*Mycobacterium chimaera* infections subsequent to cardiac surgery are related to contaminated heater-cooler devices, with high mortality. Nevertheless, few studies have been reported in Asia.

**Case presentation:**

We described the case of a 55-year-old man with *Mycobacterium chimaera* infection following cardiac surgery in the mainland of China. He was diagnosed with endocarditis caused by *Mycobacterium chimaera* subsequent to open heart surgery. Metagenomic next-generation sequencing (mNGS) and 16S rRNA gene PCR analysis were used to identify potential pathogens. The patient underwent redo valve replacement surgery and received combination therapy with azithromycin, ethambutol, linezolid, and amikacin. No signs of relapse were observed during the 11-month follow-up visit.

**Conclusions:**

This is the first documented case of *Mycobacterium chimaera* infection following cardiac surgery in the mainland of China and the first documented transnational imported case worldwide. Moreover, mNGS is a novel diagnostic technology that can guide antimicrobial therapy prior to obtaining fluid/tissue culture results for *Mycobacterium chimaera*, providing a new approach for the detection of potential *Mycobacterium chimaera* infection.

## Background

*Mycobacterium chimaera* has been described as an opportunistic pathogen, that is capable of causing human infections. Several studies have indicated that *Mycobacterium chimaera* infections following cardiac surgery are associated with aerosols from contaminated Stockert 3T heater-cooler devices [1, 2], and have a high mortality rate of 46–63% [2, 3]. *Mycobacterium chimaera* infections after cardiac surgery were initially reported by Achermann et al. in Switzerland in 2013 [4], followed by an epidemic outbreak worldwide, but has rarely been reported in Asia.

## Case presentation

A 55-year-old man who suffered from chest tightness was admitted to our hospital in September 2019. In November 2016, he was diagnosed with severe aortic valve insufficiency and underwent aortic valve replacement with a mechanical prosthetic valve at a hospital in Spain. At the time, the patient denied that he had undergone any other invasive procedure before the surgery. Nevertheless, in March 2019, he was hospitalized due to cough and fever for 1 week. Echocardiography revealed mechanical prosthetic valve perivalvular leakage,which did not abate after 2 weeks of antibiotic therapy. Later, the patient was transferred to a hospital in Shanghai China, where his symptoms were relieved after treatment with linezolid and methylprednisolone.

**Fig. 1:**
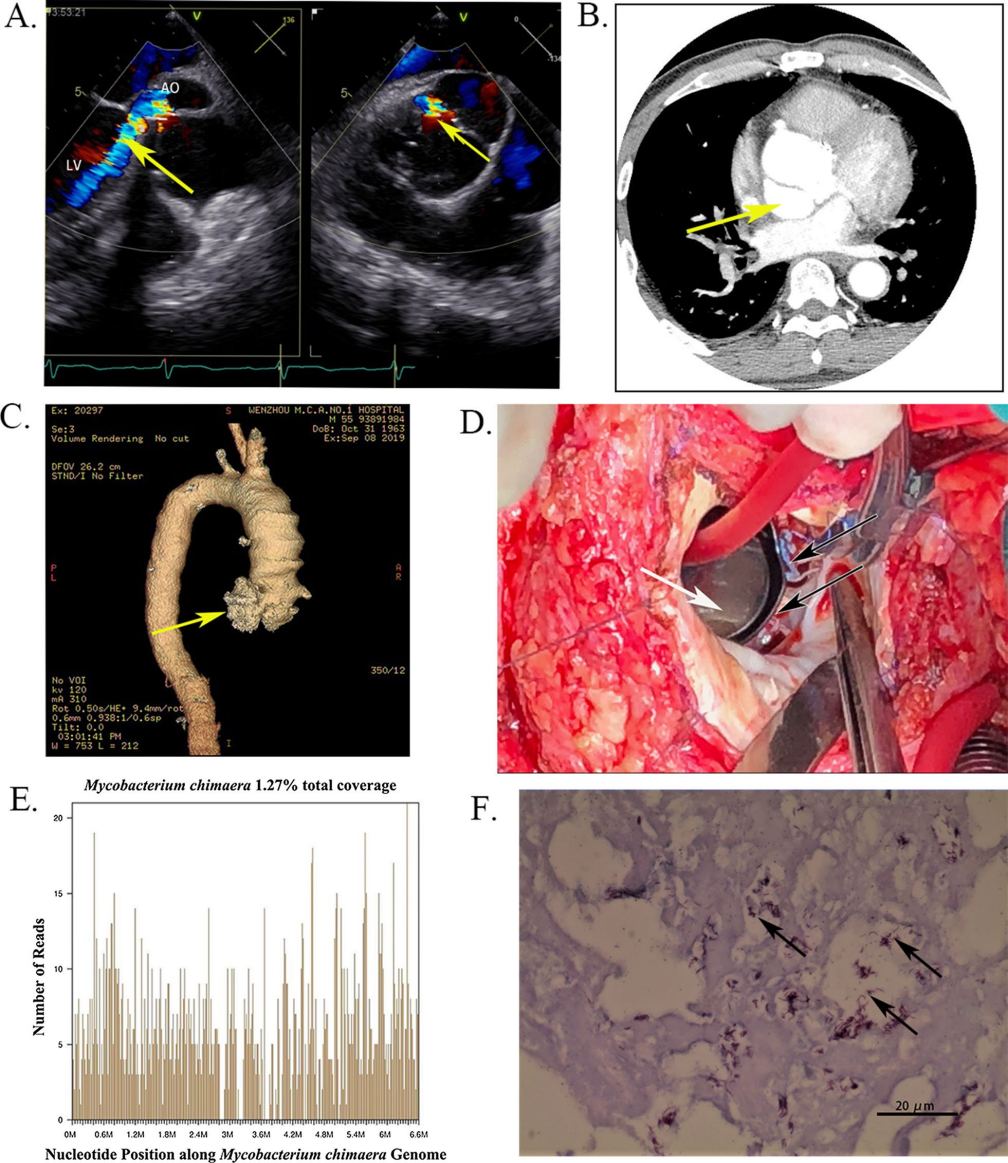
**A** Transesophageal echocardiography (TOE) of the patient. The arrows indicate severe perivalvular leakage of the aortic mechanical prosthetic valve. **B**, **C** Cardiac computed tomographic angiography (CTA) of the patient. Arrows indicate fistula of the noncoronary cusp annulus. **D** Periprosthetic fistulas connecting the left ventricle and aorta (black arrow) and the previous aortic mechanical prosthetic valve (white arrow). **E** Genome coverage of detected sequences of *Mycobacterium chimaera*. **F** The result of histopathological examination. Arrows show positive Zeihl–Neelsen acid-fast staining results

In September 2019, on admission to our hospital, his physical examination revealed a diastolic murmur in the aortic valve area, without any other abnormal physical findings. Subsequently, transesophageal echocardiography (TOE) showed severe perivalvular leakage of the aortic valve (Fig. 1A), and computed tomographic angiography (CTA) of the ascending aorta indicated periprosthetic fistulas (Fig. 1B,C). Blood culture was performed 3 times, but no predominant pathogen was discovered. The patient was diagnosed with endocarditis subsequent to open heart surgery, without pulmonary involvement.

The patient underwent redo valve replacement surgery on September 3rd, 2019. During the surgery, obvious periprosthetic fistulas connecting the left ventricle and aorta were noticed in both the left coronary cusp (LCC) annulus and the noncoronary cusp (NCC) annulus (Fig. 1D). In addition, necrotic tissues and inflammatory tissues were found in the fistulas, which were sampled for bacterial culture and histopathological examination. Unbiased metagenomic next-generation sequencing (mNGS) was adopted to detect pathogens, and artificial dural tablets were utilized to cover and suture fistulas cavities. The original aortic mechanical prosthetic valve functioned well, but was still replaced to isolate the bacterial infection.

After the surgery, a total of 27,437,693 high-quality sequencing reads were generated by mNGS on September 20th, 2019, of which 35,975 reads could be aligned to microorganisms in the reference database. As shown in Fig. 1E, a total of 1975 reads were aligned to *Mycobacterium chimaera*, with a genome coverage of 1.27%. Bacterial colonies were found via bacterial culture of the tissue on October 23rd, 2019. Then,16 S rRNA gene PCR revealed that the sequence was identical to that of *Mycobacterium chimaera* (GenBank No.NR_029003.1). In addition, the histopathological examination results showed necrotic tissue with hyaline degeneration, and Zeihl–Neelsen acid-fast staining results were positive (Fig. 1F).

The negative blood culture results, and the positive mNGS, prolonged tissue culture, and the histopathological examination results indicated that *Mycobacterium chimaera* was the pathogen responsible for the infection, and also excluded the possibility of other pathogens. Then, combination antimicrobial therapy (amikacin, azithromycin, ethambutol and linezolid), rather than empirical antibiotic therapy (combined treatment with ceftriaxone, imipenem and cilastatin sodium), was used to treat the *Mycobacterium chimaera* infection. Finally, the patient’s clinical symptoms including chest tightness, fever and coughing were relieved. No signs of relapse were observed during the 11-month follow-up visit.

## Discussion and conclusions

Due to its non-specific symptoms and insidious onset, the diagnosis of *Mycobacterium chimaera* infection is difficult, and sometimes it is misdiagnosed, leading to glucocorticoid abuse and increased systematic dissemination rate [5]. One recent study has found that non-specific symptoms such as fever, initially appear from 6 weeks to 5 years after cardiac surgeries [3]. In our case, the patient developed initial symptoms 28 months after cardiac surgery, and was subsequently treated with methylprednisolone. Six months later, he was correctly diagnosed and treated appropriately when it progressed to complicated periprosthetic fistulas.

Imaging techniques are of great significance in the diagnosis of *Mycobacterium chimaera* infection. Transthoracic echocardiography (TTE) is preferred for patients suspected of having *Mycobacterium chimaera* infection following cardiac surgery. Several researches have clarified that TOE is more sensitive than TTE [2]. Cardiac CTA or positron emission tomography computed tomography (PET-CT) can also be considered for diagnosis, especially when TTE or TOE shows negative results [6]. In the present study, TTE, TOE, and cardiac CTA were used to determine the exact sites of the infection in this patient.

It is essential to identify pathogens when considering cardiac infection. In a study conducted by Scriven et al., *Mycobacterium chimaera* was cultured in fluids/tissues, and they found that the overall diagnostic sensitivity of a single mycobacterial blood culture was 68% prior to initiation of therapy, but decreased to 34% at the start of treatment [3]. Additionally, molecular diagnosis is widely applied to identify pathogens of cardiac infections [7]. In our case, three sets of blood cultures were negative, and sample of the infected area was sent for culture during redo valve replacement surgery. However, because of the slow growth of *Mycobacterium chimaera*, ranging from 2 to 100 weeks [3], another sample of the infected area was sent for mNGS for quick results. Two weeks after surgery, mNGS indicated that *Mycobacterium chimaera* was the pathogen. Seven weeks after surgery, the sequences of cultured bacterial colonies were completely consistent with the results of the 16 S rRNA gene PCR test. Histopathological examination results showed inflammation, necrotic tissue and positive Zeihl–Neelsen acid-fast staining, which further confirmed the presence of *Mycobacterium chimaera* infection [8].

By 2019, more than 100 cases of *Mycobacterium chimaera* infection had been reported worldwide, especially in Europe and the United States [9]. The patient in our case underwent cardiac surgery in Spain. To our knowledge, this is the first documented case in the mainland of China and the first documented transnational imported case worldwide.

Because of the long duration of the disease progression and non-specific symptoms, it is difficult to avoid the delay and misdiagnosis of *Mycobacterium chimaera* infection. Therefore, enhanced surveillance to detect potential infections is critical. As a new diagnostic technology, unbiased mNGS can identify *Mycobacterium chimaera* infection before fluid/tissue culture results are available, which provides a novel method for detecting patients with potential *Mycobacterium chimaera* infection.

## Data Availability

Data and materials are fully available in the manuscript text and in the figure.
